# Dual DNA binding mode of a turn-on red fluorescent probe thiazole coumarin

**DOI:** 10.1371/journal.pone.0239145

**Published:** 2020-09-17

**Authors:** Sudakshina Ganguly, Debasis Ghosh, Nagarjun Narayanaswamy, T. Govindaraju, Gautam Basu

**Affiliations:** 1 Department of Biophysics, Bose Institute, Kolkata, India; 2 Bioorganic Chemistry Laboratory, New Chemistry Unit, Jawaharlal Nehru Centre for Advanced Scientific Research, Bengaluru, India; CIC bioGUNE, SPAIN

## Abstract

Turn-on fluorescent probes show enhanced emission upon DNA binding, advocating their importance in imaging cellular DNA. We have probed the DNA binding mode of thiazole-coumarin (TC) conjugate, a recently reported hemicyanine-based turn-on red fluorescent probe, using a number of biophysical techniques and a series of short oligonucleotides. TC exhibited increased fluorescence anisotropy and decreased absorbance (~50%) at low [DNA]/[TC] ratio. Although the observed hypochromicity and the saturating value of [DNA base pair]:[TC] ratio is consistent with a previous study that suggested intercalation to be the DNA binding mode of TC, a distinctly different and previously unreported binding mode was observed at higher ratios of [DNA]:[TC]. With further addition of DNA, only oligonucleotides containing A_n_T_n_ or (AT)_n_ stretches showed further change—decreased hypochromicity, red shifted absorption peaks and concomitant fluorescence enhancement, saturating at about 1:1 [DNA]: [TC]. ^1^H-NMR chemical shift perturbation patterns and H1’-H6/H8 NOE cross-peaks of the 1:1 complex indicated minor groove binding by TC. ITC showed the 1:1 DNA binding event to be endothermic (*ΔH°* ~ 2 kcal/mol) and entropy driven (*ΔS°* ~ 32 cal/mol/K). Taken together, the experimental data suggest a dual DNA binding mode by TC. At low [DNA]/[TC] ratio, the dominant mode is intercalation. This switches to minor groove binding at higher [DNA]/[TC], only for sequences containing A_n_T_n_ or (AT)_n_ stretches. Turn-on fluorescence results only in the previously unreported minor groove bound state. Our results allow a better understanding of DNA-ligand interaction for the newly reported turn-on probe TC.

## Introduction

DNA is a ubiquitous biological macromolecule whose specific detection under various conditions forms the basis of probing molecular mechanisms behind various cellular processes [1]. A typical double helical DNA molecule can bind appropriate molecular partners at the major or the minor groove. The binding partner can also intercalate between stacked base pairs. The major groove is much wider than the minor groove and typically accommodates protein domains. Small molecules, both natural and synthetic, display either minor groove binding or intercalation [2].

The most versatile way of mapping molecular architecture of a cell is through fluorescence-detected imaging [3]. Typically, a small molecule is designed such that it binds specifically to a particular type of cellular component or molecule and shows fluorescence only in the bound state. They include minor groove binders, like Hoechst dyes or quinone cyanine dithiazole [4, 5], or intercalators, like ethidium bromide (EtBr) [6] or propidium iodide (PI) [7]. Some fluorophores such as DAPI may behave as an intercalator or minor groove binder depending upon the DNA sequence [8]. Despite the availability of a variety of molecular probes, most of the commercially available fluorophores for DNA staining suffer from various disadvantages. DAPI and Hoechst dyes require ultraviolet light for excitation that may cause photo-induced DNA damage due to the production of free radicals leading to cell death [9]. The impermeable character of EtBr and PI has restricted their use only to fixed and dead cells. Cyanine dyes have also been used for staining of DNA gels and fluorescence based assays but these dyes do not display a high degree of specificity and also result in enhanced fluorescence with RNA and single stranded DNA (ssDNA) [10].

**Fig 1:**
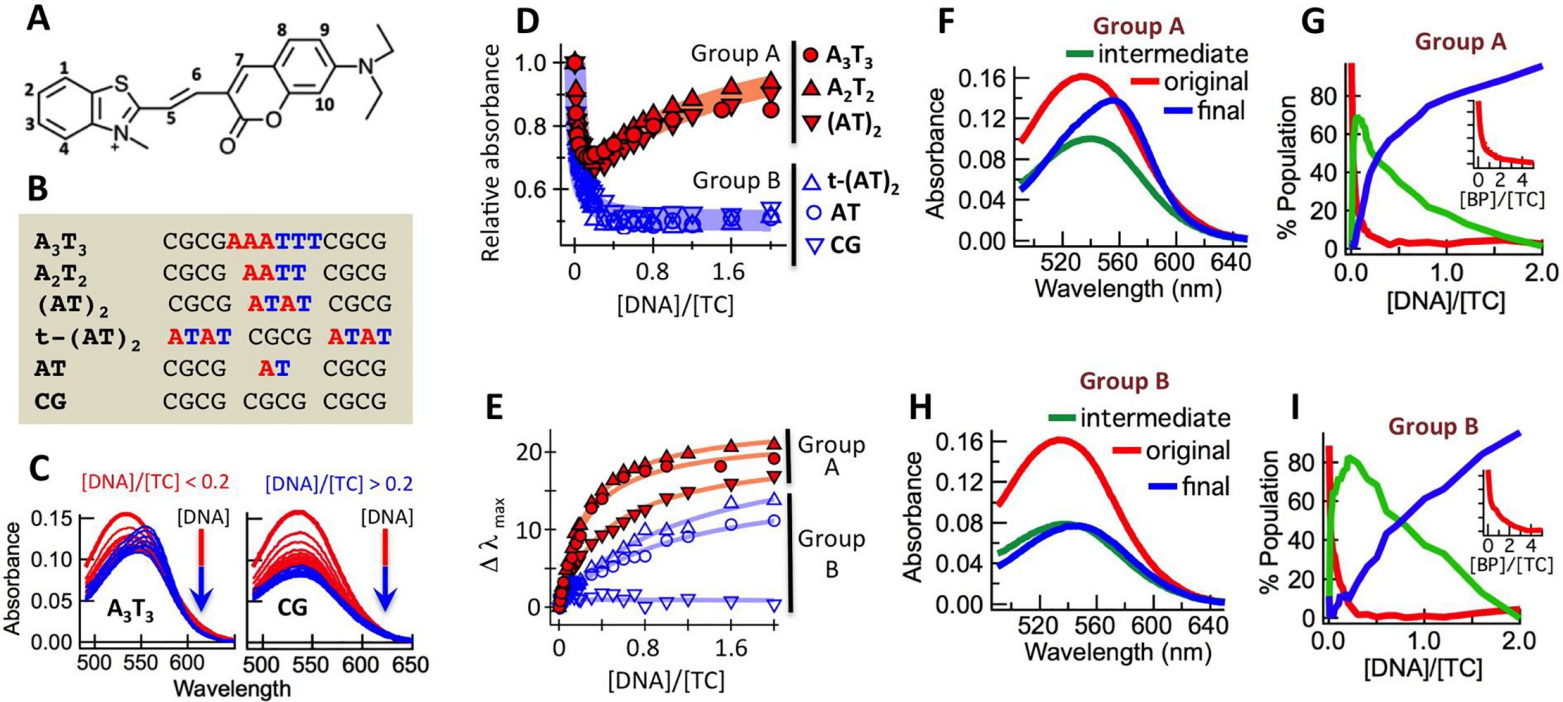
**A.** Molecular structure of TC. **B.** Oligonucleotides used in this study. **C.** Variation of absorption spectra of TC as a function of added **A**_**3**_**T**_**3**_ (left panel) and **CG** (right panel). **D.** Changes in absorbance of TC (5 μM) as a function of increasing [DNA]/[TC]. **E.** Changes in absorption maxima of TC (5 μM) as a function of increasing [DNA]/[TC]. **F.** CCA-derived average basis spectra derived from DNA-titrated (with **A**_**3**_**T**_**3**_, **A**_**2**_**T**_**2**_ and **(AT)**_**2**_) absorption spectra of TC (S2 Fig). **G.** Percent population of the basis spectra (panel F) as a function of [DNA]/[TC]. Inset shows % population of the original species as a function of [base pair]/[TC]. **H.** CCA-derived average basis spectra derived from DNA-titrated (with **AT** and t-**(AT)**_**2**_) absorption spectra of TC (S2 Fig). **I.** Percent population of the basis spectra (panel H) as a function of [DNA]/[TC]. Inset shows % population of the original species as a function of [base pair]/[TC].

An ideal fluorescent probe should have certain attributes- excitation at longer wavelengths to prevent auto fluorescence of the cellular components, ability to show turn-on fluorescence in presence of dsDNA, high permeability to cellular membranes, low levels of toxicity to live cells, high specificity to dsDNA over ssDNA or RNA and responsive at low concentrations. The design of a recently reported hemicyanine-based fluorescent probe, thiazole-coumarin conjugate (TC; Fig 1A), takes into account these criteria [11]. The turn-on red fluorescent TC showed specificity towards AT-base pairs in dsDNA.

Although preliminary experiments, based on FRET assays with Hoechst 33258, and DFT calculations suggested that TC binds DNA via intercalation, the DNA binding mode by TC was not probed directly. Here we have studied the DNA-binding mode of TC to a number of systematically designed short oligonucleotides containing AT or GC stretches (Fig 1B) using a number of complementary biophysical techniques. We show that TC, in addition to intercalation, binds at the minor groove of AT-rich DNA–a binding mode responsible for the fluorescence enhancement.

## Materials and methods

### Materials

In total, six self-complementary oligonucleotides were used in this study: (1) **A**_**2**_**T**_**2**_: 5’-(CG)_2_A_2_T_2_(CG)_2_−3’; (2) **A**_**3**_**T**_**3**_: 5’-(CG)_2_A_3_T_3_(CG)_2_−3’; (3) **(AT)**_**2**_: 5’-(CG)_2_(AT)_2_(CG)_2_−3’; (4) **t-(AT)**_**2**_: 5’-(AT)_2_(CG)_2_(AT)_2_−3’; (5) **AT**: 5’-(CG)_2_AT(CG)_2_−3’; (6) **CG**: 5’-(CG)_6_−3’. All oligonucleotides (Fig 1B) were purchased from GCC Biotech in HPLC purified form and dissolved in buffer A (10 mM sodium phosphate, 10 mM NaCl, pH 7.5). The oligonucleotides were annealed by heating to 90 ºC for 5 minutes followed by slow cooling overnight. TC was synthesized and purified as described elsewhere [11]. The experimental procedures followed for the synthesis of TC, and associated analytical data, are included in the Supplementary Materials (S1 Text and S1 Fig).

### Electronic spectroscopy

The UV-visible spectra of TC, with and without DNA, were recorded on a Shimadzu UV-1700 spectrophotometer in buffer A. Spectra obtained from titrations were deconvoluted using the method of Convex Constrained Analysis (CCA) using the software CCA+ [12] where the spectrum of TC in absence of DNA was used as one of the basis spectra. Fluorescence spectra of TC, with and without DNA, were recorded on a PTI QM-400 fluorimeter (λ_ex_ = 530 nm) in buffer A. Fluorescence anisotropy was measured using a L-format configuration. Unless otherwise stated, all experiments were performed at 25°C with [TC] = 5 μM. Binding constants were estimated using

$$F(L)=F_{0}+(F_{\infty }-F_{0})[K_{d}+n+LC_{0}-\sqrt{{\left(K_{d}+n+LC_{0}\right)}^{2}-4nC_{0}L}]/2nC_{0}$$ (1)

where *F, F*_0_ and (*F*_∞_−*F*_0_) are the observed, initial and final (under saturating conditions) fluorescence, *n* is the number of binding sites, *C*_0_ and *L* are the receptor and ligand concentrations, respectively.

### Isothermal Titration Calorimetry (ITC)

ITC experiments were performed on a TA Affinity ITC instrument. Samples were extensively degassed prior to the experiment, conducted in buffer A and at 25°C. Titrations were performed by injecting incremental amounts of TC into **A**_**3**_**T**_**3**_ (40 μM). The interval between two subsequent injections and the injection volume were 200 seconds and 2 μl, respectively. The observed heat rates were corrected by subtracting heat of dilution obtained by repeating the experiment without **A**_**3**_**T**_**3**_ (only buffer A). The thermograms were analysed using the in-built NanoAnalyze software.

### NMR spectroscopy

NMR spectra were acquired on Bruker Avance-III 700 MHz spectrometer in presence of 10% D_2_O (for signal locking) and trimethyl-silyl propionate (internal standard). TC:DNA titrations were performed by recording 1D ^1^H spectra upon incremental addition of either TC or DNA to the other species (300 μM in buffer A). Resonance assignments and proximity of protons were determined from 2D TOCSY and NOESY (mixing time = 250 ms) experiments recorded under similar conditions [13]. Unless otherwise stated, all experiments were performed at 25°C.

### Molecular docking

Molecular docking was performed with Autodock 4.0 [14] using 2DAU (pdb ID) as the DNA template. The 3D structure of TC was generated using Corina [15] and used for docking after energy minimization.

## Results

### UV-Vis absorption spectroscopy

The absorption spectra of TC were recorded as a function of increasing amounts of added oligonucleotides (S2 Fig). AT-rich (**A**_**3**_**T**_**3**_) and GC-rich (**CG**) DNA induced very different changes in the absorption spectra of TC (Fig 1C). As shown in Fig 1D, in terms of DNA-induced changes in the absorption spectra, the oligonucleotides could be classified into two distinct groups (group A: **A**_**3**_**T**_**3**_, **A**_**2**_**T**_**2**_ and **(AT)**_**2**_; group B: **t-(AT)**_**2,**_ **AT** and **CG**).

For group A, the absorbance decreased initially before increasing again while group B showed monotonic decrease. Addition of DNA also resulted in red shifts (Fig 1E): highest for **A**_**3**_**T**_**3**_ and **A**_**2**_**T**_**2**_ (~ 20 nm), intermediary for **(AT)**_**2**_, **t-(AT)**_**2**_ and **AT** (~ 10–15 nm) and no detectable shift for **CG**. The titration-derived UV-vis spectra of TC were subjected to CCA, a method that deconvolutes a set of spectra as a linear combination of two or more basis spectra.

As shown in S3 Fig, CCA yielded three basis spectra (initial, intermediate and final) for all except **CG** (for which two basis spectra were obtained). The average basis spectra and their growth or decay are shown in Fig 1F and 1G (group A) and Fig 1H and 1I (group B). Compared to the initial basis spectra, the intermediate basis spectra showed reduced intensity (37% reduced for group A and 50% reduced for group B) and red shift (5 nm for group A and 3 nm for group B). Appearance of the intermediary species was completed by [DNA]/[TC] ~ 0.2 ([base pair]/[TC] ~ 3; inset to Fig 1G and Fig 1I) after which the final species started to build up with increasing DNA. Compared to the intermediary species, the final species for group A showed large red shift (15 nm) and enhanced absorbance (40%). For group B the final basis spectrum showed a small red shift (7 nm) with no change in absorbance.

### Fluorescence spectroscopy

**Fig 2:**
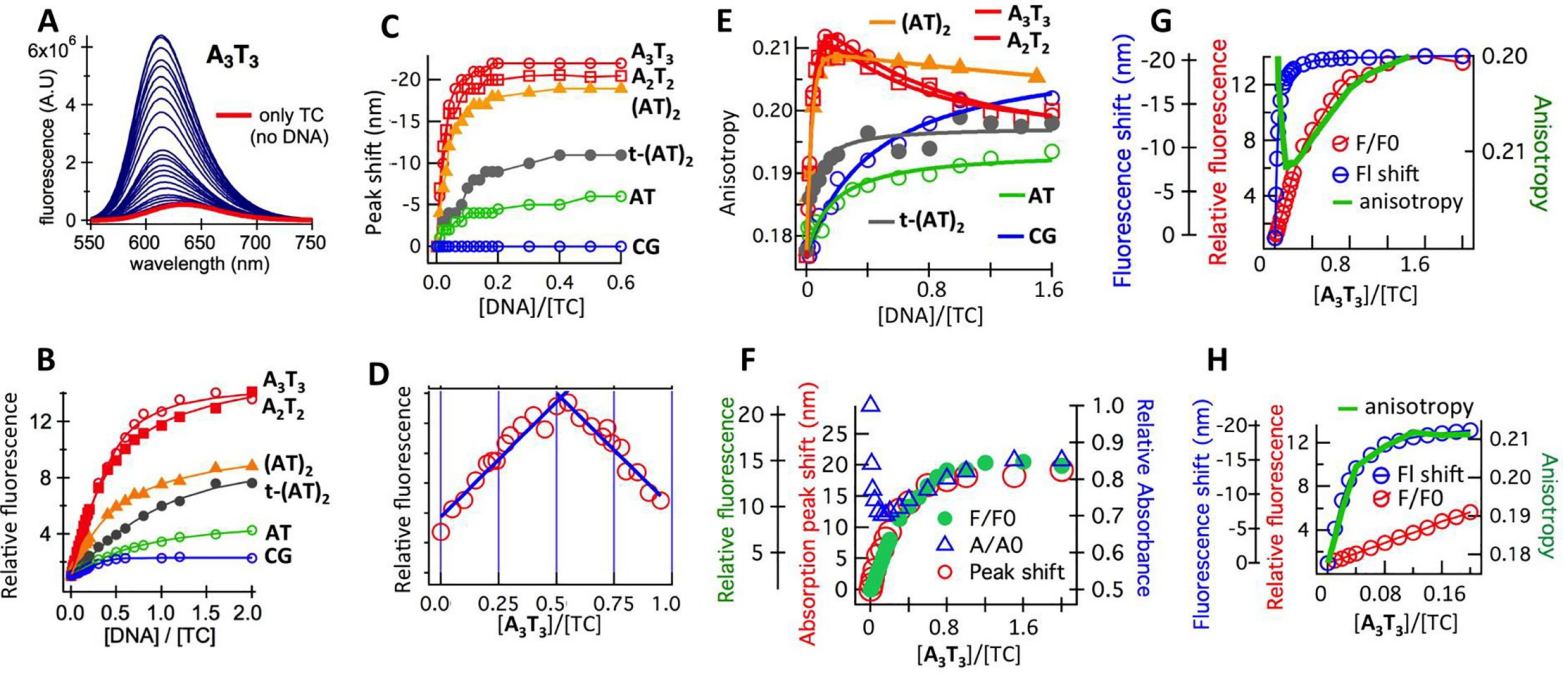
**A.** Fluorescence spectra of TC as a function of added **A**_**3**_**T**_**3**_. **B.** Relative fluorescence enhancement (corrected for altered absorbance; see Fig 1D) of TC as a function of added DNA. **C.** Fluorescence peak shifts of TC as a function of added DNA. **D.** Job plot obtained from fluorescence enhancement of TC at different [**A**_**3**_**T**_**3**_]/[TC] ratios. **E.** Fluorescence anisotropy of TC as a function of added DNA. **F.** Overlay of fluorescence enhancement, absorption peak shift and relative absorbance of TC as a function of [**A**_**3**_**T**_**3**_]/[TC]. **G.** Overlay of fluorescence peak shift, fluorescence enhancement and fluorescence anisotropy of TC as a function of [**A**_**3**_**T**_**3**_]/[TC]. **H.** Expanded panel G (up to [**A**_**3**_**T**_**3**_]/[TC] = 0.2).

The fluorescence spectra of TC were recorded as a function of increasing amounts of added oligonucleotides (Fig 2A and S4 Fig). As shown in Fig 2B, **A**_**3**_**T**_**3**_ and **A**_**2**_**T**_**2**_ showed the highest fluorescence enhancement (~ 12-fold), enhancement by **(AT)**_**2**_ and **t-(AT)**_**2**_ were moderate (~ 8-fold) while fluorescence enhancement by **AT** and **CG** was nominal. The members of group A showed large blue shift (~ 20 nm), **t-(AT)**_**2**_ and **AT** from group B showed intermediary blue shift (~ 5–10 nm) while the addition of **CG** did not produce any peak shift (Fig 2C). The blue shifts were completed by [DNA]/[TC] ~ 0.2 while fluorescence enhancement saturated around [DNA]/[TC] ~ 1.0, consistent with a 1:1 **A**_**3**_**T**_**3**_:TC binding stoichiometry obtained from fluorescence intensity detected Job plot (Fig 2D).

The fluorescence anisotropy of TC, as a function of added DNA, was also measured (Fig 2E). The anisotropy of free TC (~ 0.18) increased initially upon addition of **A**_**3**_**T**_**3**_ or **A**_**2**_**T**_**2**_ (for [DNA]/[TC] < 0.2) and then decreased before finally stabilizing at [DNA]/[TC] ~ 1.2. **(AT)**_**2**_ also showed similar trend. However, anisotropy increase for members of group B was smaller and the increase was monotonic. Changes in fluorescence and absorbance were correlated for **A**_**3**_**T**_**3**_ (Fig 2F): fluorescence enhancement coincided with absorption peak shift and the increase in absorbance (observed for [DNA]/[TC] > 0.2). Changes in fluorescence (intensity and peak shift) was also correlated with changes in anisotropy: the gradual decrease in anisotropy ([DNA]/[TC] > 0.2) correlated with the increase in fluorescence intensity (Fig 2G) while the sharp increase in anisotropy ([DNA]/[TC] < 0.2) correlated with the fluorescence blue shift (Fig 2H).

### NMR spectroscopy

**Fig 3:**
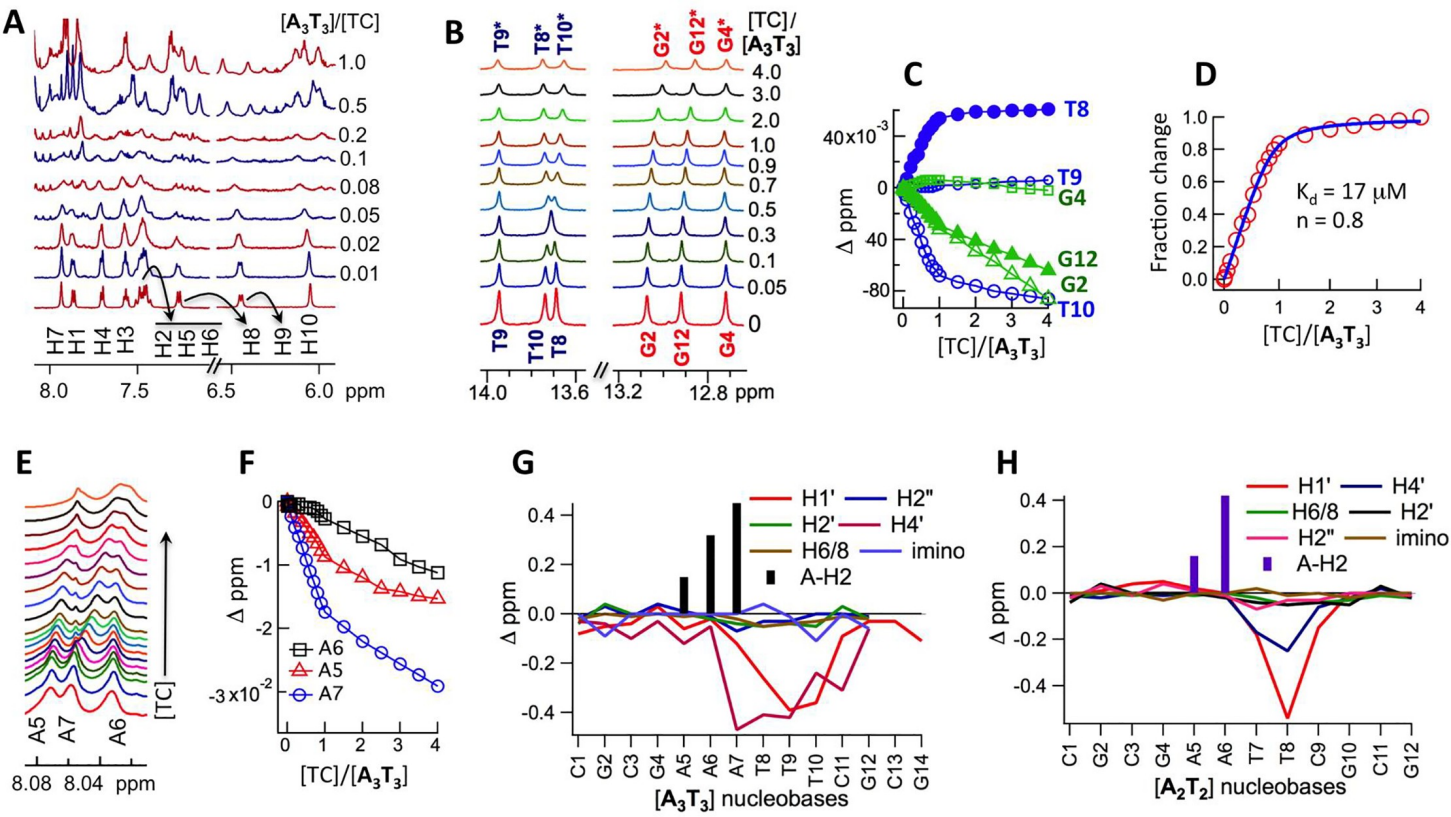
**A.** ^1^H resonances of TC (300 μM) as a function of added **A**_**3**_**T**_**3**_. **B.** ^1^H imino resonances of **A**_**3**_**T**_**3**_ (300 μM) as a function of added TC. **C.** Chemical shift variation of imino protons (panel B) as a function of [TC]/[**A**_**3**_**T**_**3**_]. **D.** Average fraction change (chemical shifts) of T8 and T10 imino protons (panel C) as a function of [TC]/[**A**_**3**_**T**_**3**_]. The solid line represents the best fit with a binding model (Eq 1). **E.** ^1^H resonances (H8) of adenine in **A**_**3**_**T**_**3**_ (300 μM) as a function of added TC. **F.** Chemical shift variation of H8 protons (panel E) as a function of [TC]/[**A**_**3**_**T**_**3**_]. **G.** Chemical shift perturbation between free and 1:1 **A**_**3**_**T**_**3**_:TC complex. **H.** Chemical shift perturbation between free and 1:1 **A**_**2**_**T**_**2**_:TC complex.

The ^1^H NMR spectrum of TC showed sharp and distinct peaks in solution (Fig 3A) that could be assigned using TOCSY experiments. The sharp resonances underwent downfield shift and broadening upon addition of DNA (Fig 3A and S5A Fig). By ~ [**A**_**3**_**T**_**3**_]/[TC] = 0.2, TC resonances almost disappeared due to broadening. When the reverse titrations were performed (TC added to **A**_**3**_**T**_**3**_) TC peaks did not reappear even at [TC]/[**A**_**3**_**T**_**3**_] = 4, although considerable shifts were observed for the DNA (**A**_**2**_**T**_**2**_ and **A**_**3**_**T**_**3**_) protons.

Due to absence of resonance overlap with TC, the imino resonances of **A**_**3**_**T**_**3**_ (Fig 3B) and **A**_**2**_**T**_**2**_ (S5B Fig) were monitored as a function of added TC (the imino resonances of **CG** were also monitored as control; see S5C Fig). For **A**_**3**_**T**_**3**_ (Fig 3C), T9 and G4 showed almost no change. Unlike G2 and G12, by [**A**_**3**_**T**_**3**_]/[TC] = 1, changes in T8 and T10 resonances were almost complete. That this change corresponded to TC:DNA binding was reflected in the excellent fit of the fractional change in chemical shifts of T8 and T10 to Eq 1 (Fig 3D). Chemical shifts of the relatively overlap-free H8 resonance of A5, A6 and A7, bases complimentary to T10, T9 and T8, respectively, showed similar trend (Fig 3E and 3F). Chemical shifts of A6 changed almost linearly while those of A5 and A7 showed a bimodal change (with a break at [**A**_**3**_**T**_**3**_]/[TC] ~ 1).

The difference of DNA ^1^H chemical shifts, between TC-bound (1:1 complex) and TC-free state, are shown in Fig 3G (**A**_**3**_**T**_**3**_) and Fig 3H (**A**_**2**_**T**_**2**_). In both cases the biggest change was observed at the center, from A5 to C11 in **A**_**3**_**T**_**3**_ and from A5 to C9 in **A**_**2**_**T**_**2**_. The chemical shift perturbation patterns showed large downfield shifts for the adenine H2 protons and large upfield shifts for the sugar H1’ and H4’ protons, which line the floor and walls of the minor groove. The corresponding major groove protons, including aromatic H6/8 and H2’, experienced much smaller shifts upon TC addition.

### DNA binding thermodynamics

**Fig 4:**
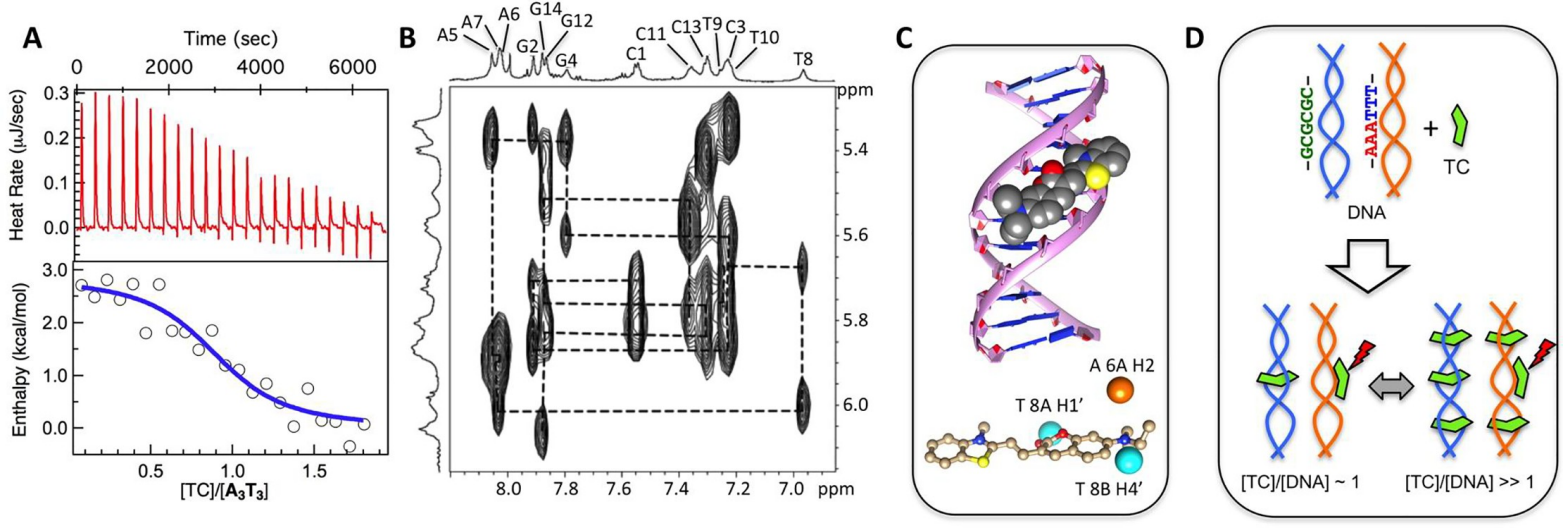
**A.** ITC profile (heat rates corrected for heat of dilution versus time and enthalpy versus [TC]/[DNA]) of TC added to **A**_**3**_**T**_**3**_ at 25°C. **B.** H1’-H6/H8 NOE cross-peaks of 1:1 **A**_**3**_**T**_**3**_:TC complex. **C.** Docked structure TC with DNA (**A**_**2**_**T**_**2**_). As shown in the bottom, H1’ and H4’ protons of T8 are equatorial and H2 proton of A6 is axial to TC in the docked structure. **D.** A schematic model depicting modes of DNA binding by TC: at low [TC]/[DNA], TC intercalates GC-rich DNA and binds the minor groove of AT-rich DNA. At high [TC]/[DNA] AT-rich DNA show dual DNA binding modes: intercalation and minor groove binding.

The thermodynamics of the binding was directly monitored calorimetrically (from ITC experiments), by adding TC to **A**_**3**_**T**_**3**_, and the relevant thermodynamic parameters were directly extracted by fitting the data to a single site binding model (Fig 4A). This yielded: ΔH° = 2.0 kcal/mol, ΔS° = 32.1 cal/mol/K, K_d_ = 2.9 μM and n = 0.94. The entropic nature of the binding was also evident when the fluorescence enhancement of TC was monitored as a function of **A**_**3**_**T**_**3**_ at different temperatures. Subsequent data fitting at each temperature (to Eq 1) yielded n < 1, indicating the binding to be complex (S6 Fig). Nevertheless, temperature dependence of K_d_ (**A**_**3**_**T**_**3**_ added to TC) indicated the binding event to be endothermic in nature (ΔH° = 5.6 kcal/mol and ΔS° = 19 cal/mol/K from a van’t Hoff analysis; S6 Fig).

It is known that van’t Hoff analysis can be prone to errors [16]. Therefore, for our analysis we will only focus on the qualitative conclusion from the van’t Hoff analysis—that the binding is endothermic and entropically driven. On the other hand, the more trustworthy and robust calorimetric values of ΔH° and ΔS° will be used for any quantitative analysis.

## Discussion

Design of new molecules that exhibit turn-on fluorescence can add substantial power to DNA visualizing techniques. However, to fully understand the mechanism of fluorescence enhancement and the origin of sequence specificity, the DNA binding mode that triggers fluorescence must be understood. Using complementary biophysical techniques and six short dsDNA, we have probed the DNA binding modes of TC [11], a recently reported turn-on red fluorescent probe.

Consistent with the earlier report, DNA sequences containing A_n_T_n_ stretches (**A**_**2**_**T**_**2**_ and **A**_**3**_**T**_**3**_) showed maximum fluorescence, (AT)_n_ stretches (**(AT)**_**2**_ and **t-(AT)**_**2**_) showed intermediary or low fluorescence enhancement while **CG** (with no A/T) showed almost no fluorescence enhancement. Job Plot suggested a 1:1 binding stoichiometry for **A**_**3**_**T**_**3**_. Yet, upon closer examination, it was clear that the binding was more complex.

Although the fluorescence enhancement saturated at [DNA]/[TC] ~ 1 for AT-rich DNA, the associated peak shifts saturated at much less [DNA]:[TC] ratio (~ 0.2). Like the peak shift, anisotropy (Fig 2E) also showed saturation at [DNA]/[TC] ~ 0.2. The differential saturation of complimentary fluorescence parameters clearly indicated two different modes of DNA:TC interaction for AT-rich DNA, operating at two different [DNA]/[TC] ratios. The modes of interaction at two [DNA]:[TC] ratios were also sequence-dependent, as evident from the sequence-dependence of the extent of fluorescence enhancement, peak shift and anisotropy. Binding heterogeneity was also reflected in the value of n (stoichiometry of DNA molecules bound to TC) which was consistently < 1 when fluorescence enhancement was fitted to Eq 1 (S6 Fig; Fig 2B).

The presence of the dual DNA binding mode of TC, dependent on [DNA]/[TC] values, was also apparent from the UV-vis absorption data. The low [DNA]/[TC]-regime interaction manifested itself in the abrupt appearance of hypochromicity. CCA showed that three slightly different intermediary species were generated by three different DNA groups during this mode: (i) **A**_**3**_**T**_**3**_, **A**_**2**_**T**_**2**_ and **(AT)**_**2**_ (ii) **t-(AT)**_**2**_ and **AT** (iii) **CG**. While the UV-vis spectra of all intermediate species showed reduced absorbance, the extent of the associated red shift was sequence-dependent (no shift for **CG**). The absorbance signature for the other DNA interaction mode, that turns on beyond [DNA]/[TC] ~ 0.2, was the loss of the initial hypochromicity and further red shift. While this was true for **A**_**3**_**T**_**3**_, **A**_**2**_**T**_**2**_ and **(AT)**_**2**_, **t-(AT)**_**2**_ and **AT** showed a different signature: the hypochromicity remained but absorption spectra showed a small red shift. The absorption spectra of **CG**, on the other hand, showed no significant change beyond [DNA]/[TC] ~ 0.2.

To understand the nature of the binding mode at low [DNA]/[TC] we focus on the remarkable hypochromicity (~ 50%) associated with this binding mode. Hypochromicity is known to be characteristic of π-stacked molecules [17]. For our case this could indicate end stacking or DNA intercalation. With 12-mer oligonucleotides used in our study, end stacking would require ~ 6 base pairs per TC molecule. The average number of base pairs needed for intercalation, on the other hand, is ~ 3 [18]. The DNA-binding event at low [DNA]/[TC] is complete by [base pair]/[TC] ~ 3 for all six DNA (insets to Fig 1G and Fig 1I), strongly suggesting intercalation to be the DNA-binding mode at low [DNA]/[TC]. A simple end stacking model can also be ruled out since it would have made **t-(AT)**_**2**_ stand out from the other five DNA that contain GC base pairs at the termini, which was not observed. Further support for intercalation comes from the earlier report [11] where FRET was observed between TC and Hoechst 33258 (a minor groove binder) when TC was added to Hoechst 33258-bound (AT)_20_. Intercalation was also reported to be energetically favourable from DFT calculations.

We now look at the other binding event, dominating beyond [DNA]/[TC] ~ 0.2. As shown by ITC, NMR and Job Plot, this event yielded a 1:1 TC:DNA complex for **A**_**3**_**T**_**3**_, **A**_**2**_**T**_**2**_ and **(AT)**_**2**_. Since all sequential connectivities between the sugar H1’ and the aromatic H6/8 protons of the next nucleotide in TC-bound DNA (1:1) could be readily assigned for **A**_**2**_**T**_**2**_ and **A**_**3**_**T**_**3**_ (Fig 4B and S7 Fig), intercalation can be ruled out as the second binding mode. On the other hand, chemical shift perturbation patterns clearly pointed towards minor groove binding. For **A**_**2**_**T**_**2,**_ the H1’ and H4’ protons of T8 were equatorial to TC while the H2 proton of A6 were axial to TC in a minor groove docked model of TC:**A**_**2**_**T**_**2**_ (Fig 4C). The equatorial and axial placements are in agreement with the observed upfield shifts of H1’/H4’ and downfield shifts of H2 (in both **A**_**2**_**T**_**2**_ and **A**_**3**_**T**_**3**_). The docked model lacks any specific DNA-TC interaction like H-bonds which can enthalpically stabilize the bound state. This is compatible with the endothermic and entropy driven nature of TC: **A**_**3**_**T**_**3**_ interaction, as observed from ITC and van’t Hoff analysis. Interestingly, it is this 1:1 minor groove bound TC:DNA complex that exhibited the functionally important enhanced fluorescence for **A**_**3**_**T**_**3**_, **A**_**2**_**T**_**2**_ and (**AT)**_**2**_. For **t-**(**AT)**_**2**_ and **AT**, the 1:1 TC:DNA complex exhibited only medium fluorescence enhancement. For **CG**, the second binding mode seemed to be absent.

The low [DNA]/[TC] regime binding event was also characterized by extreme broadening of TC peaks in the ^1^H NMR spectrum. Just like the observed hypochromicity associated with this event, the line broadening was observed for AT-rich **A**_**3**_**T**_**3**_ (Fig 3A) / **A**_**2**_**T**_**2**_ (S5A Fig) and AT-devoid (**CG**) sequences (S5D Fig). The line broadening can be attributed to intermediate exchange between free TC and DNA-TC complex. In fact, intermediate exchange has been reported to be the reason behind extreme line broadening of ligand peaks for certain cyanine dyes [19].

Taken together, the experimental data presented above suggest a model of DNA binding by TC (Fig 4D) that depends on both DNA sequence and [TC]/[DNA] ratio. For sequences with only CG stretches, mode of DNA interaction by TC is intercalation at all [TC]/[DNA] values. If the DNA sequence is AT-rich (either A_n_T_n_ or (AT)_n_ stretches) the dominant DNA binding mode at [TC]/[DNA] ~1 is minor groove binding. At [TC]/[DNA] >> 1, in addition to minor groove binding, TC can also intercalate DNA. However, if the AT-stretch is at the termini (like in **t-**(**AT)**_**2**_) or if it is only two-nucleotide long (like in **AT**) a mixed binding mode (intercalation and incomplete groove binding) ensues at [TC]/[DNA] ~ 1 while intercalation is the dominant mode at [TC]/[DNA] >> 1.

The coumarin moiety, an integral part of TC, is known to intercalate DNA [20]. So it is not surprising that TC was found to intercalate DNA. However, with the added thiazole group, the longer TC molecule containing a positive charge is better suited for minor groove binding. AT stretches provide the ideal minor groove pocket for this to be accomplished. As a result, TC shows a dual DNA binding mode with AT-rich sequences but remains confined to intercalating DNA if the sequence is GC rich. Dual DNA binding modes have been shown for some other well-known DNA binders [21, 22] and TC becomes a new addition. What is unique about TC is that it shows fluorescence enhancement only when bound in the minor groove. This probably happens due to conformational restriction over the length of the molecule, when inserted in the minor groove. Intercalation would not facilitate this if only the coumarin moiety lodges in the intercalation site.

## Supporting information

S1 Text Synthetic procedure for TC. (PDF)

S1 Fig ^1^H-NMR, ^13^C-NMR and HRMS spectrum of TC. (JPG)

S2 Fig Absorption spectra of TC as a function of added DNA. (JPG)

S3 Fig CCA analysis of absorption spectra of TC as a function of added DNA. (JPG)

S4 Fig Fluorescence spectra of TC as a function of added DNA. (JPG)

S5 Fig ^1^H resonances of TC as a function of added A_2_T_2_ / CG and ^1^H resonances of A_2_T_2_ /CG as a function of added TC. (JPG)

S6 Fig The fluorescence titration of TC (5 μM) with A_3_T_3_ at different temperatures and the associated van’t Hoff plot. (JPG)

S7 Fig H1’-H6/H8 NOE cross-peaks of A_2_T_2_ (in 1:1 complex with TC). (JPG)

## References

[1] Travers A, Muskhelishvili G. DNA structure and function. FEBS J. 2015; 282: 2279–2295. 10.1111/febs.13307 25903461

[2] Bhaduri S, Ranjan N, Arya DP. An overview of recent advances in duplex DNA recognition by small molecules. Beilstein J Org Chem. 2018; 14: 1051–1086. 10.3762/bjoc.14.93 29977379 PMC6009268

[3] Suseela YV, Narayanswamy N, Pratihar S, Govindaraju T. Far-red fluorescent probes for canonical and noncanonical nucleic acid structures: current progress and future implications. Chem Soc Rev. 2018; 47; 1098–1131. 10.1039/c7cs00774d 29264610

[4] Feixue H, Taulier N, Chalikian TV. Association of the Minor Groove Binding Drug Hoechst 33258 with d(CGCGAATTCGCG)_2_: Volumetric, Calorimetric and Spectroscopic Characterizations. Biochemistry 2005; 44: 9785–9794. 10.1021/bi047374f 16008363

[5] Narayanaswamy N, Das S, Samanta PK, Banu K, Sharma GP, Mondal N, et al Sequence-specific recognition of DNA minor groove by an NIR-fluorescence switch-on probe and its potential applications. Nucleic Acids Res. 2015; 43: 8651–8663. 10.1093/nar/gkv875 26350219 PMC4605319

[6] Nafisi S, Saboury AA, Keramat N, Neault JF, Tajmir-Riahi HA. Stability and structural features of DNA intercalation with ethidium bromide, acridine orange and methylene blue. J Mol Struct. 2007; 827: 35–43.

[7] Banerjee A, Majumder P, Sanyal S, Singh J, Jana K, Das C, et al The DNA intercalators ethidium bromide and propidium iodide also bind to core histones. FEBS Open Bio. 2014; 4: 251–259. 10.1016/j.fob.2014.02.006 24649406 PMC3958746

[8] Kubota Y, Kubota K, Tani S. DNA binding properties of DAPI (4',6-diamidino-2-phenylindole) analogs having an imidazoline ring or a tetrahydropyrimidine ring: groove-binding and intercalation. Nucleic Acids Symp Ser. 2000; 44: 53–54. 10.1093/nass/44.1.53 12903264

[9] Pfeifer GP, You YH, Besaratinia A. Mutations induced by ultraviolet light. Mutat Res. 2005; 571; 19–31. 10.1016/j.mrfmmm.2004.06.057 15748635

[10] Zipper H, Brunner H, Bernhagen J, Vitzthum F. Investigations on DNA intercalation and surface binding by SYBR Green I, its structure determination and methodological implications. Nucleic Acids Res. 2004; 32: e103 10.1093/nar/gnh101 15249599 PMC484200

[11] Narayanaswamy N, Kumar M, Das S, Sharma R, Samanta PK, Pati SK, et al A thiazole coumarin (TC) turn-on fluorescence probe for AT-base pair detection and multipurpose applications in different biological systems. Sci Rep. 2014; 4: 6476 10.1038/srep06476 25252596 PMC4174567

[12] Perczel A, Hollósi M, Tusnády G, Fasman GD. Convex constraint analysis: a natural deconvolution of circular dichroism curves of proteins. Protein Eng. 1991; 4: 669–679. 10.1093/protein/4.6.669 1946324

[13] Wuthrich K. NMR of Proteins and Nucleic Acids. Wiley, New York 1986. ISBN: 978-0-471-82893-8

[14] Morris GM, Huey R, Lindstrom W, Sanner MF, Belew RK, Goodsell DS, et al Autodock4 and AutoDockTools4: automated docking with selective receptor flexiblity. J. Comput. Chem. 2009; 16: 2785–91. 10.1002/jcc.21256 PMC2760638 19399780

[15] Sadowski J, Gasteiger J, Klebe G. Comparison of Automatic Three-Dimensional Model Builders Using 639 X-Ray Structures. J. Chem. Inf. Comput. Sci. 1994; 34: 1000–1008.

[16] Chaires JB. Possible origin of differences between van't Hoff and calorimetric enthalpy estimates. Biophys Chem. 1997; 64:15–23. 10.1016/s0301-4622(96)02205-3 9127935

[17] Nakano T. Synthesis, structure and function of π-stacked polymers. Polym J. 2010; 42: 103–123

[18] Stockert JC. A neighbour-exclusion intercalating model for poly(ADP-ribose) binding to DNA. J. Theor Biol. 1983; 105: 461–467. 10.1016/0022-5193(83)90187-x 6656289

[19] Hannah KC, Gil RR, Armitage BA. ^1^H NMR and optical spectroscopic investigation of the sequence-dependent dimerization of a symmetrical cyanine dye in the DNA minor groove. Biochemistry 2005; 44: 15924–15929. 10.1021/bi051298e 16313195

[20] Amin KM, Taha AM, George RF, Mohamed NM, Elsenduny FF. Synthesis, antitumor activity evaluation, and DNA‐binding study of coumarin‐based agents. Arch Pharm Chem Life Sci. 2018; 351: e1700199. 10.1002/ardp.201700199 29148081

[21] Trotta E, D'Ambrosio E, Ravagnan G, Paci M. Simultaneous and different binding mechanisms of 4',6-diamidino-2-phenylindole to DNA hexamer (d(CGATCG))_2_. A 1H NMR study. J Biol Chem. 1996; 271: 27608–14. 10.1074/jbc.271.44.27608 8910349

[22] Larsson A, Carlsson C, Jonsson M, Albinssont B. Characterization of the Binding of the Fluorescent Dyes YO and YOYO to DNA by Polarized Light Spectroscopy. J Am Chem Soc. 1994; 116: 8459–8465.

