# Supplementary material for: Dual DNA binding mode of a turn-on red fluorescent probe thiazole coumarin
Source: PLoS One. 2020 Sep 17;15(9):e0239145. doi: 10.1371/journal.pone.0239145 (PMC7497988; doi:10.1371/journal.pone.0239145)
Supplement: S1 Text — (PDF) [file pone.0239145.s001.pdf]

S1 Text. Synthetic procedure for TC.

To a stirred solution of 2-methyl benzothiazole (7.0 mmol) in dichloromethane (10 mL), methyl iodide (14.0 mmol) was added drop wise and allowed to reflux for overnight. Completion of the reaction was monitored with TLC. After completion of the reaction white color precipitate was formed. The precipitate was filtered and washed with diethyl ether for removing unreacted benzothiazole. The obtained product N-methylated benzothiazole was dried under vacuum and used for the next reaction without further purification.

To a stirred solution of N-methylated benzothiazole (0.35g, 1.2 mmol) in ethanol, piperidine (10 µl) was added and the reaction mixture was allowed to stir for 10 min. 7-(Diethylamino)-2-oxo-2H-chromene-3-carbaldehyde (0.17 g, 0.69 mmol) in ethanol solution was added dropwise to the above reaction mixture, leading to immediate yellow to purple color change in the solution after which the reaction mixture was allowed to stir for 4 h. Completion of the reaction was monitored by thin layer chromatography (TLC). After completion of the reaction, the solvent was evaporated under vacuum. The crude product was purified using column chromatography on silica gel using $CHCl_3$/MeOH as an eluent to afford TC in good yield.

Characterization data for **TC**

Brown color powder, yield 50%. ^{1}H-NMR (*DMSO-d_6*, 400 MHz) δ 8.59 (s, 1H), 8.37 (dd, J = 0.8 Hz, J = 8Hz, 1H), 8.21 (d, J = 8.4 Hz, 1H), 8.02 (dd, J = 8 Hz, J = 15.6 Hz, 2H), 7.83 (m, 1H), 7.74 (td, J = 1.2 Hz, J = 7.6 Hz, 1H), 7.57 (d, J = 9.2 Hz, 1H), 6.87 (dd, J = 2.4 Hz, J = 9.2 Hz, 1H), 6.67 (d, J = 2.4 Hz, 1H), 4.22 (s, 3H), 3.53 (q, J = 7.2 Hz, 4H), 1.17 (t, J = 7.2 Hz, 6H). ^{13}C-NMR (*DMSO-d_6*, 100 MHz) δ 171.6, 159.5, 157.1, 153.3, 148.3, 144.3, 142.0, 131.8, 129.2, 128.0, 127.4, 124.1, 116.5, 112.0, 111.3, 110.9, 108.9, 96.4, 44.6, 35.8, 12.4. HRMS (ESI-MS): calcd for $C_{23}H_{23}N_2O_2SI$ $[M-I]^+$ m/z = 391.1475, found 391.1456.

Reference: N. Narayanaswamy, M. Kumar, S. Das, R. Sharma, P. K. Samanta, S. K. Pati, S. K. Dhar3, T. K. Kundu and T. Govindaraju, A Thiazole Coumarin (TC) Turn-On Fluorescence Probe for AT-Base Pair Detection and Multipurpose Applications in Different Biological Systems, Sci. Rep. **2014**, 4, 6476, (DOI: 10.1038/srep06476).
