# Supplementary figures and images for: Dual DNA binding mode of a turn-on red fluorescent probe thiazole coumarin
Source: PLoS One. 2020 Sep 17;15(9):e0239145. doi: 10.1371/journal.pone.0239145 (PMC7497988; doi:10.1371/journal.pone.0239145)

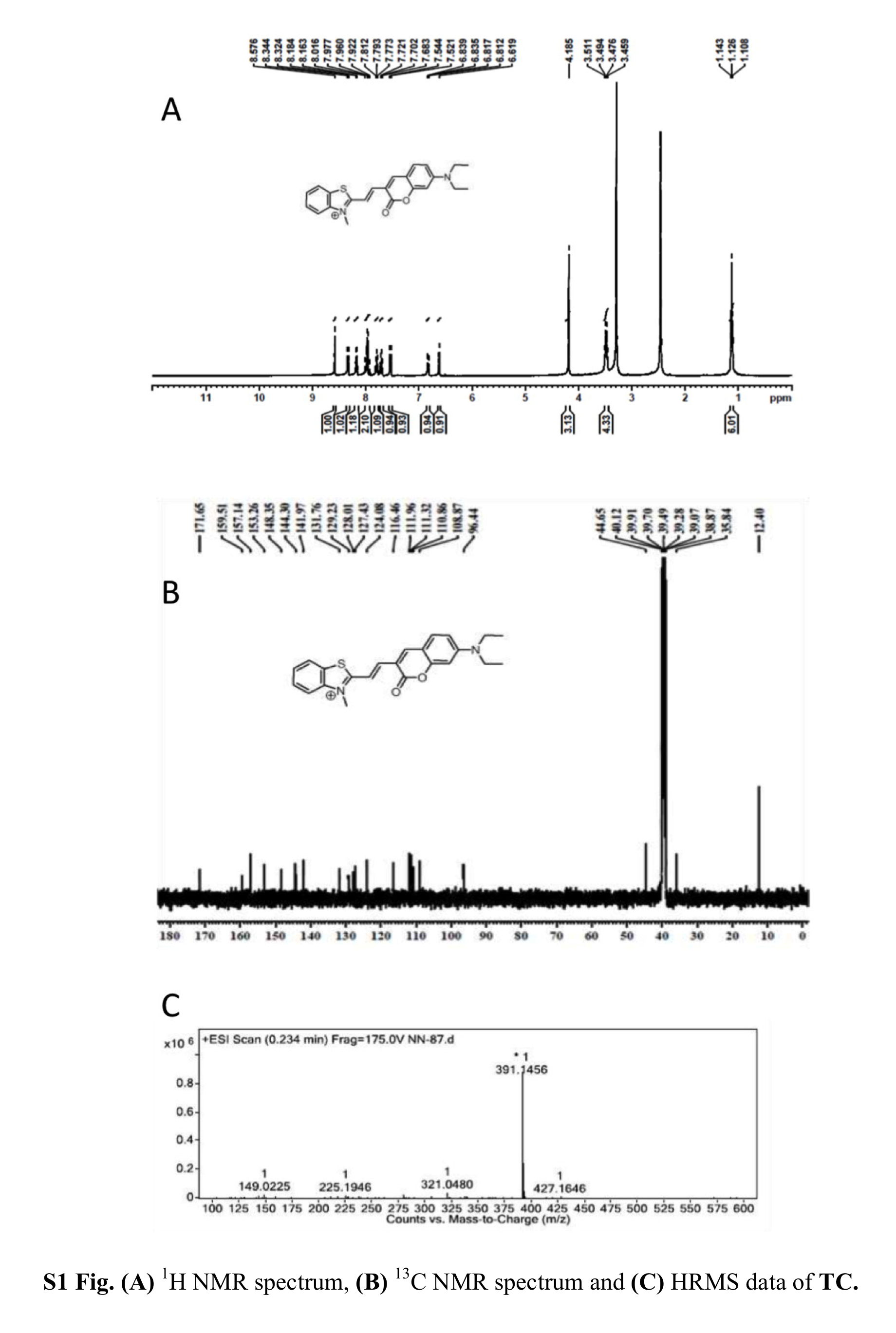

Supplement: S1 Fig — (JPG) [file pone.0239145.s002.jpg]

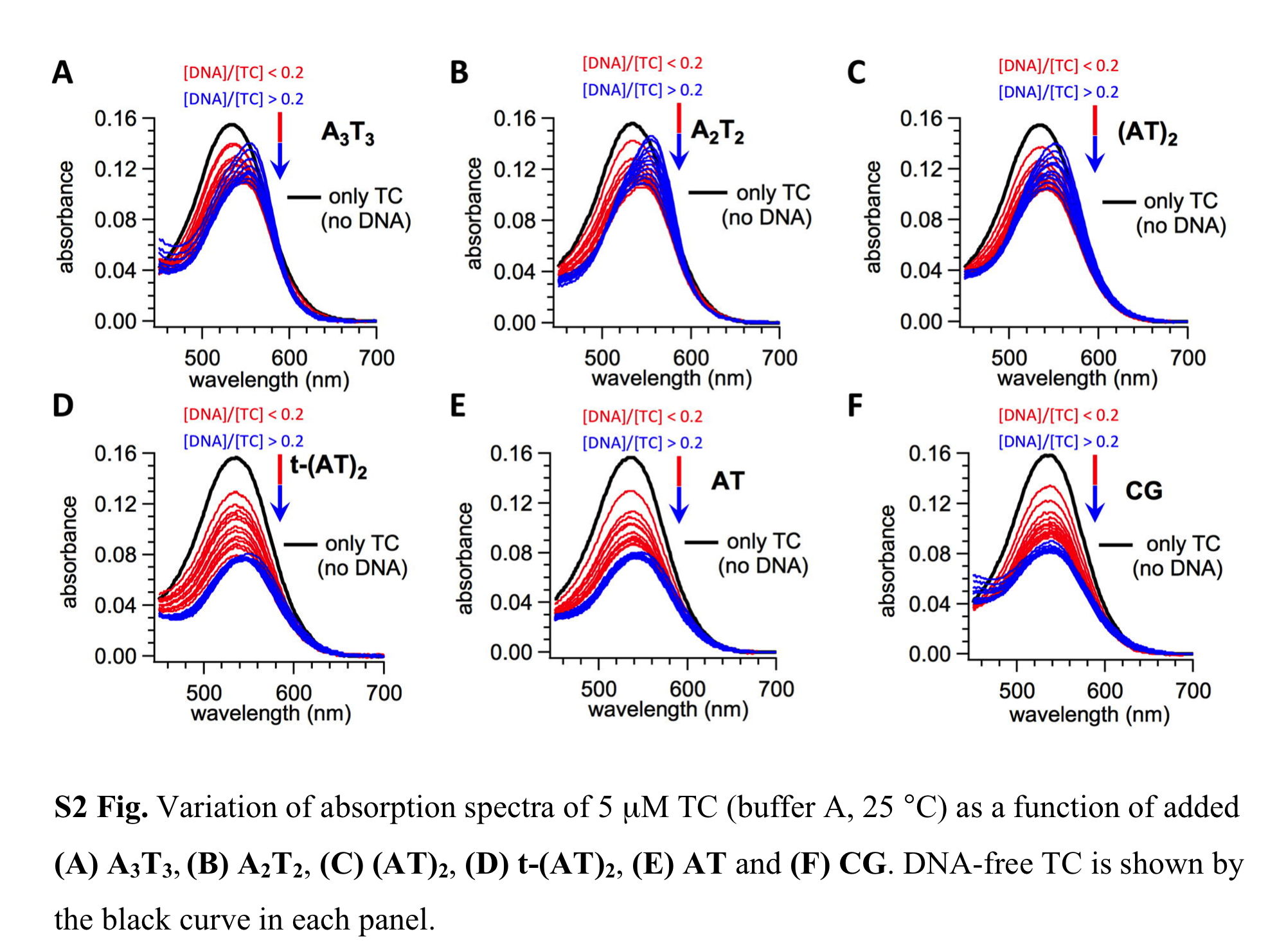

Supplement: S2 Fig — (JPG) [file pone.0239145.s003.jpg]

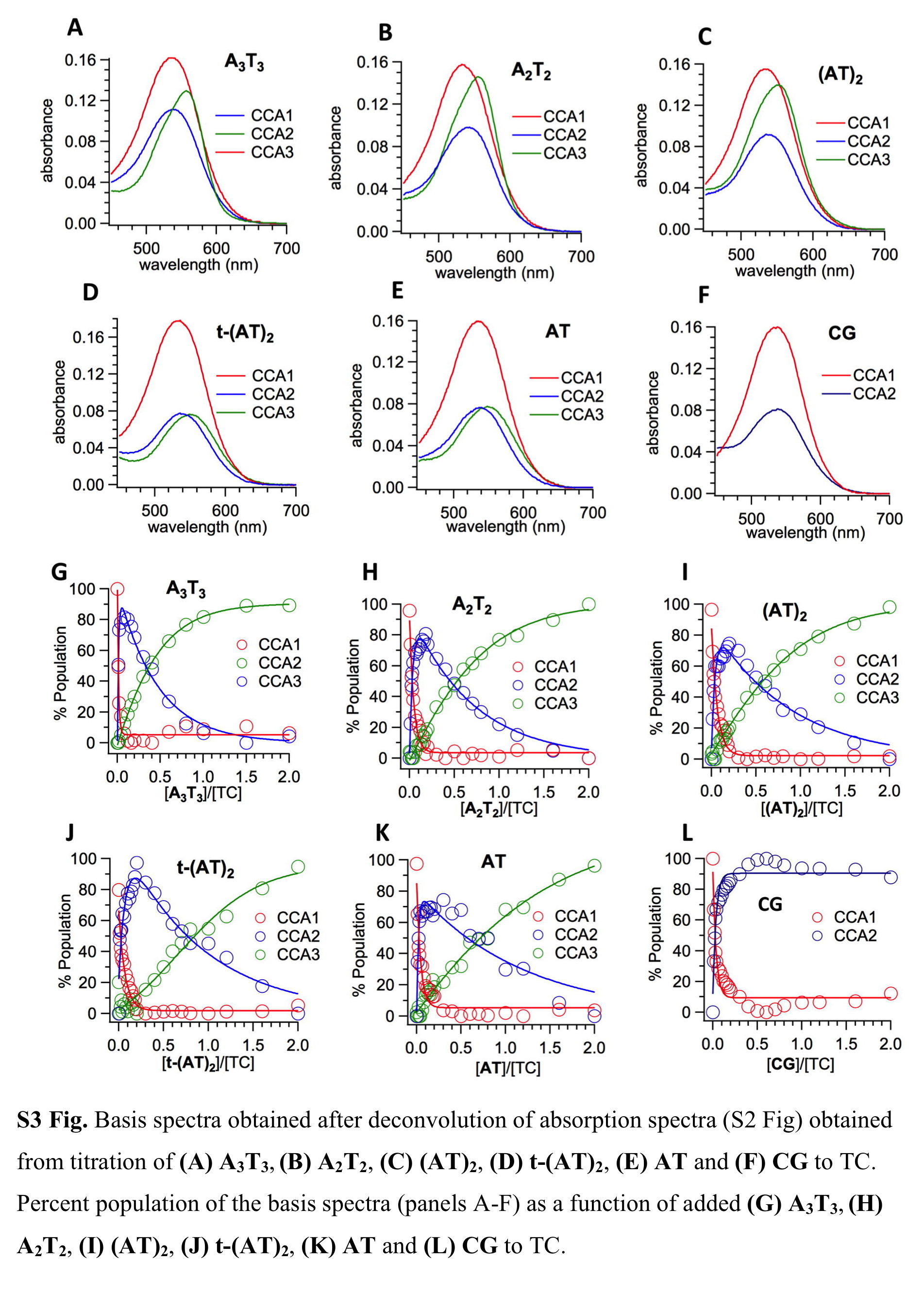

Supplement: S3 Fig — (JPG) [file pone.0239145.s004.jpg]

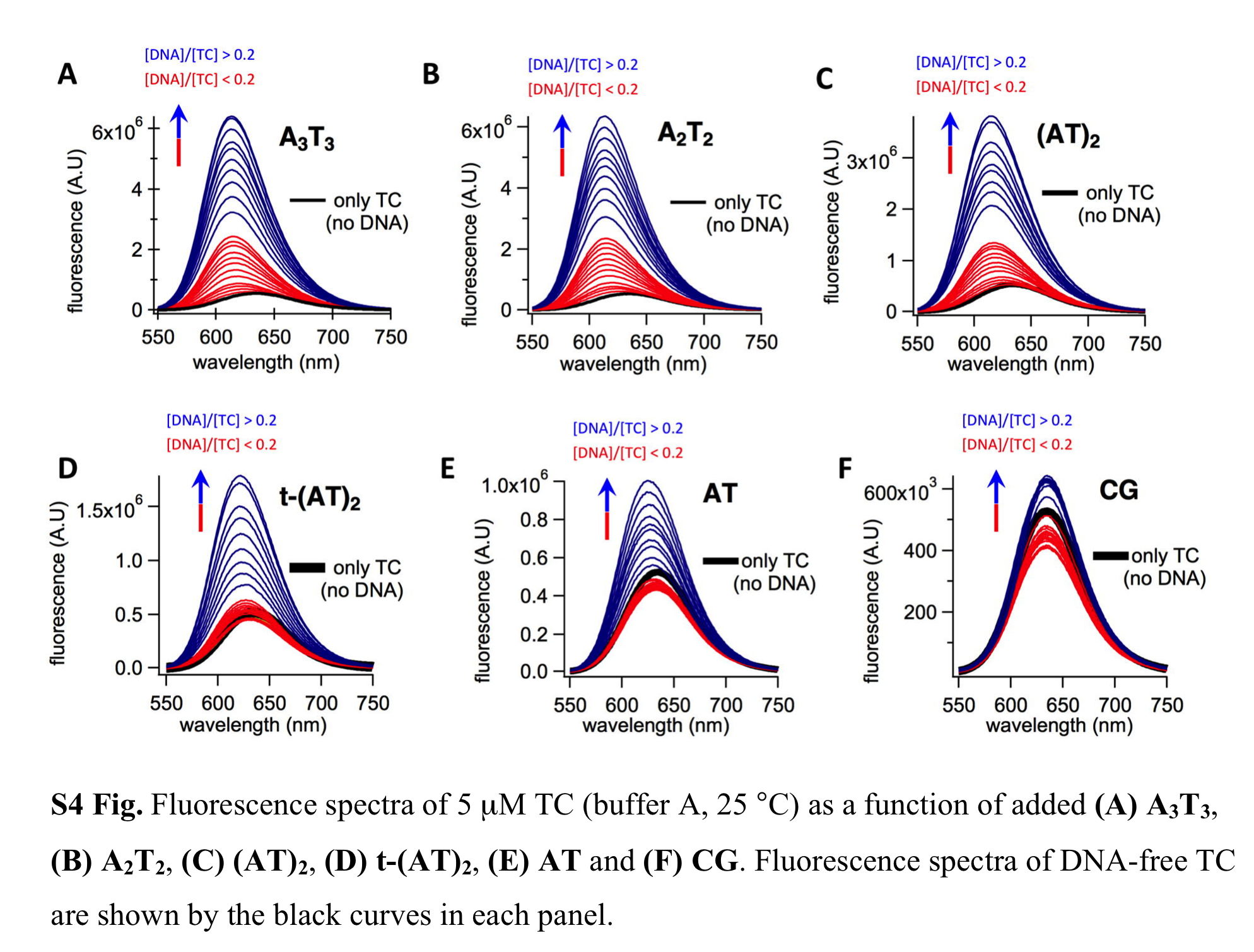

Supplement: S4 Fig — (JPG) [file pone.0239145.s005.jpg]

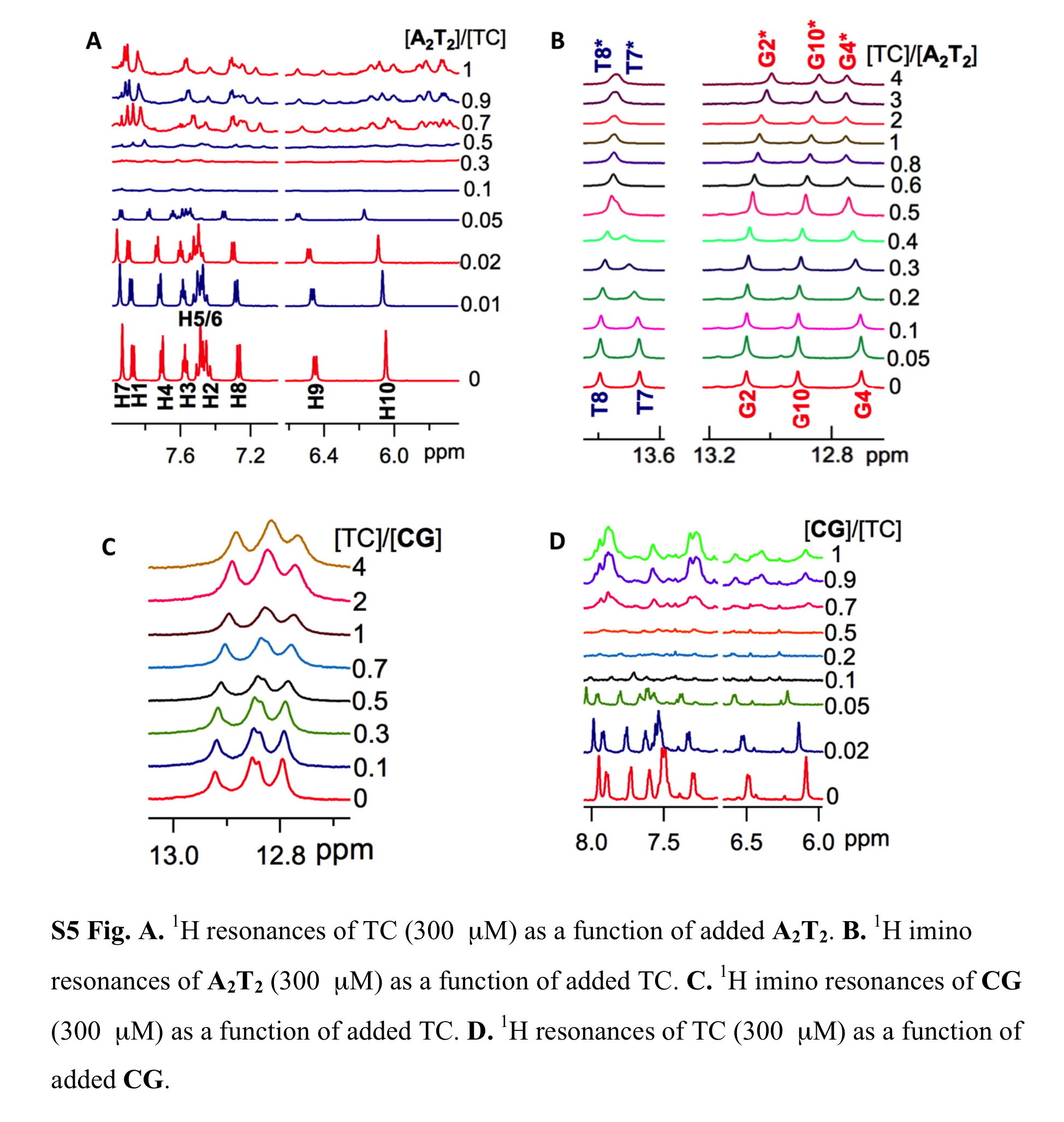

Supplement: S5 Fig — (JPG) [file pone.0239145.s006.jpg]

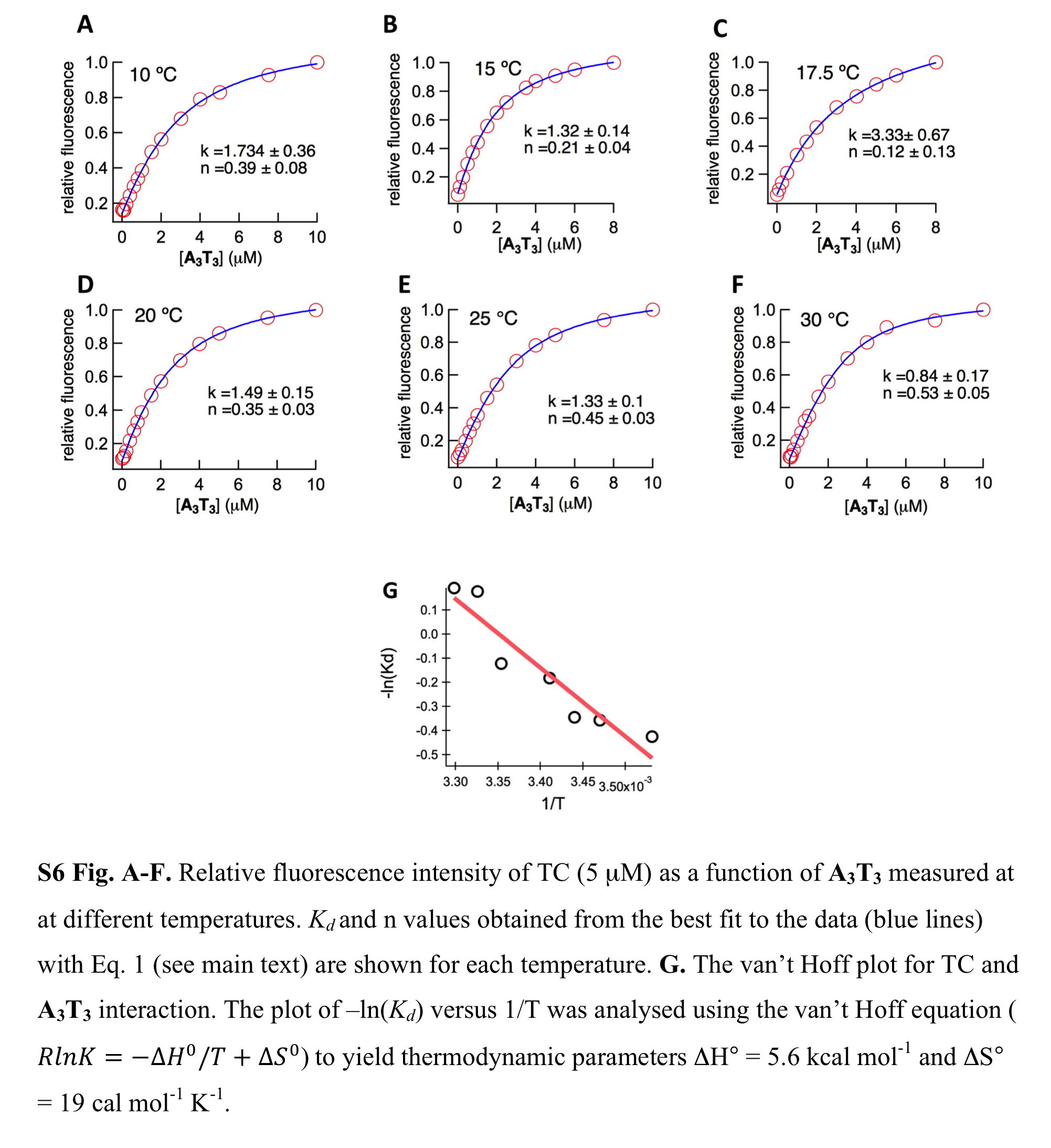

Supplement: S6 Fig — (JPG) [file pone.0239145.s007.jpg]

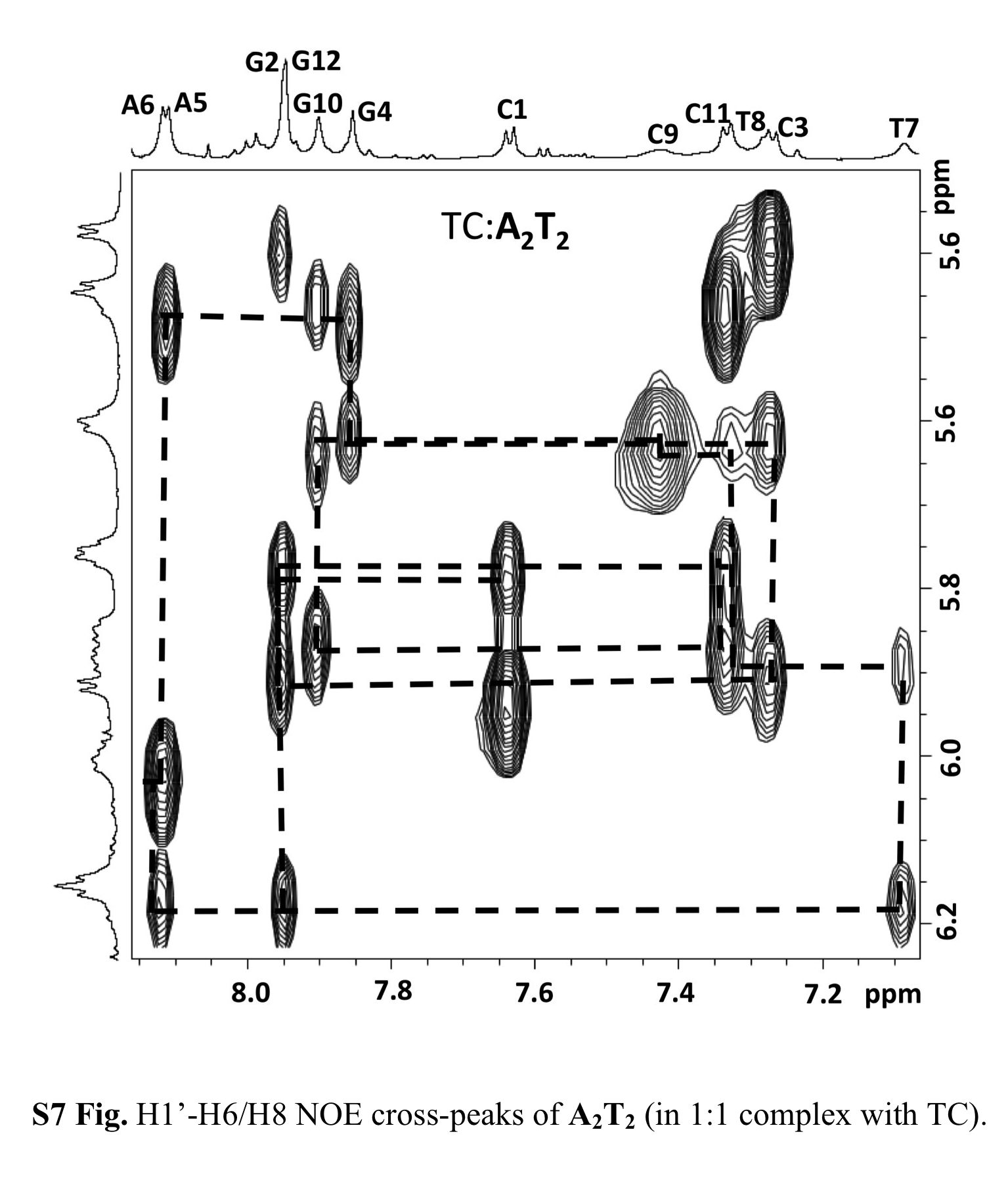

Supplement: S7 Fig — (JPG) [file pone.0239145.s008.jpg]
